# Sleep efficiency and event−related potentials in patients with depression: the mediating role of serum C-reactive protein

**DOI:** 10.3389/fpsyt.2025.1574864

**Published:** 2025-07-02

**Authors:** Liang Fang, Peng Wang, Tianqin Xie, Shuai Ding, Ting Wang, Jiajia Zhang, Aiguo Zhang, Peng Zhu, Daomin Zhu

**Affiliations:** ^1^ Department of Sleep Disorders, Affiliated Psychological Hospital of Anhui Medical University, Hefei, China; ^2^ Department of Sleep Disorders, Anhui Mental Health Center, Hefei Anhui, China; ^3^ Department of Sleep Disorders, Hefei Fourth People’s Hospital, Hefei Anhui, China; ^4^ Department of Maternal, Infant and Child Nursing, School of Nursing, Anhui Medical University, Hefei, China; ^5^ Department of Maternal, Child and Adolescent Health, School of Public Health, Anhui Medical University, Hefei, China

**Keywords:** depression, event-related potentials, sleep efficiency, chronic systemic low-grade inflammation, cognitive function

## Abstract

**Background:**

Patients with major depressive disorder (MDD) may experience cognitive dysfunction and sleep disorders. Limited research exists on the neurophysiological mechanisms that connect sleep efficiency and cognitive function in individuals with MDD. The study aims to investigate the link between sleep efficiency, mental abilities, and levels of serum C-reactive protein (CRP) in individuals diagnosed with MDD.

**Methods:**

A total of 101 individuals diagnosed with MDD were selected and categorized into two groups: the normal sleep efficiency group (NSE) with SE ≥90% and the group with lower sleep efficiency (LSE) with SE <Eth) All patients underwent polysomnography (PSG), event-related potentials (ERPs) tests, and CRP detection. The study used multiple linear regression and bootstrapped mediation analysis to explore the correlation between SE, ERPs latency, and CRP.

**Results:**

The N2, P3a, and P3b latencies were longer in the LSE group compared to the NSE group (*p* = 0.036, *p* = 0.013, *p* < 0.001). N2 (Pr = -122.182), P3a (Pr = -109.597), P3b (Pr = -151.960), and CRP (Pr = -3.768) are significantly associated with SE. A strong correlation was found between CRP (Pr = 9.414) and P3b latency. After controlling for gender and other pertinent variables, the subsequent investigation revealed a direct correlation between CRP and P3b latency, specifically within the cohort of depression patients exhibiting low SE. CRP mediated the association between SE and P3b latency.

**Conclusion:**

Low SE with MDD was associated with chronic inflammation and impaired cognitive function, suggesting that inflammation may act as a potential mediating factor in the relationship between SE and impaired cognitive function.

## Introduction

Major depressive disorder (MDD), influenced by social, psychological, and biological factors, is prevalent worldwide (1). Severe MDD can lead to decreased quality of life, impaired social functioning, cognitive impairment, neurophysiological alterations, and increased financial burden (2). Sleep disturbances affect over 80% of individuals with MDD across community and clinical populations (3). Sleep is often particularly associated with cognitive health, such as slow-wave sleep deficits that can impair cognitive function through the mechanisms that may be relevant to SE, and sleep is increasingly recognized as a modifiable risk factor for cognitive decline (4–6). Sleep disorders often accompany MDD in clinical settings, and sleeping is essential for maintaining cognitive health (4, 5). While the neurophysiological basis of cognitive dysfunction in MDD remains incompletely understood (7–10), emerging evidence implicates prefrontal cortex glutamate dysregulation in executive function and working memory deficits, along with amygdala hyperactivity, contributing to negative cognitive biases (11–13). The efficacy of existing drug and non-drug therapies remained unsatisfactory in clinical practice (14–16). Given the limited treatment options for cognitive impairment, investigating modifiable factors and underlying mechanisms becomes particularly crucial.

Event-related potentials (ERPs), particularly P3b (17, 18), are valuable for elucidating cognitive processes and detecting abnormal brain activity associated with potential disorders. Moreover, ERPs have been extensively utilized in the assessment of various neurological conditions such as dementia (19, 20) and Parkinson’s syndrome (21, 22). However, their application in research on MDD has been relatively limited (23–25). Currently, there is a lack of objective indicators (26, 27) to evaluate the relationship between cognition and sleep, primarily relying on subjective assessment questionnaires (28, 29).

Accumulating evidence shows that sleep quality has a significant impact on the regulation of inflammatory factors, which, in turn, influences overall health (30–32). C-reactive protein (CRP) is a reliable measurable acute-phase protein commonly used as an inflammation marker, indicating both peripheral and central inflammation (33, 34). A previous meta-analysis reports that individuals with major depressive disorder (MDD) who experience poor sleep patterns tend to demonstrate elevated levels of CRP (35). Recent studies indicate that MDD accompanied by sleep problems usually leads to elevated levels of inflammatory markers (31, 36, 37) as well as cognitive decline. Disrupted circadian rhythms not only heighten inflammatory responses but also contribute to the onset of neuropsychiatric disorders that adversely affect mood and cognition (30, 38, 39). In the context of cognitive decline, it is well established that inflammatory factors play a significant role (30, 31, 40). The correlation between CRP levels and sleep/cognitive indicators implies that routine CRP screening in MDD patients could potentially serve as a valuable tool to identify early cognitive impairment. While our study highlights CRP as a plausible mediator, the exact neurobiological pathways require further investigation through longitudinal imaging studies combined with cytokine profiling. We hypothesize that improving sleep quality may potentially facilitate a reduction in the body’s inflammatory response, while improvement in CRP levels could ameliorate cognitive impairment resulting from poor sleep quality. This approach holds promise for the treatment of cognitive impairment in MDD; however, there is currently limited research on this topic within MMD. It is unclear if high CRP levels affect the link between poor sleep and cognitive impairment in MDD. Future trials could stratify patients by baseline CRP levels to evaluate whether anti-inflammatory adjunct therapies yield differential benefits in sleep and cognition.

The study seeks to investigate the possible associations of sleep and serum CRP levels with cognitive function in depressed patients in a cross-sectional study. Furthermore, we aimed to explore the potential mediating role of higher CRP levels in the relationship between poorer sleep quality and cognitive decline.

## Materials and methods

### Inclusion criteria

From February 2021 to April 2023, 101 hospitalized MDD patients were recruited from the Sleep Disorders Department of Hefei Fourth People’s Hospital. During the registration process, initial screening was carried out by two or more attending physicians, using both the ICD-10 diagnostic criteria for depression and specific study criteria. The inclusion criteria included meeting the ICD-10 diagnostic criteria for depressive symptoms, age between 18 and 60 years, education level equivalent to junior high school or higher, right-handedness, and Han nationality. The exclusion criteria included the following: mental disorders such as schizophrenia, substance-induced mood disorders, anxiety disorders, bipolar disorder, and substance abuse or dependence; history of important physical diseases related to nervous system dysfunction, metabolism issues, or endocrine system abnormalities; conditions or medications known to influence systemic inflammation (like statins or NSAIDs); pregnancy status, including lactating women and those planning pregnancy; severe physical ailments or autoimmune-related problems; history of head trauma resulting in seizures lasting over 5 min, leading to consciousness disorders; auditory or sensory abnormalities; and diagnosis of atherosclerosis and/or hypertension since hypertension is linked to an elevated risk in CRP levels synergistically when considering body mass index (BMI).

Data on demographics and clinical characteristics included gender, age, education level, BMI, illness duration, smoking and alcohol habits, the utilization of benzodiazepines medications and the utilization of conventional antidepressant medications such as serotonin norepinephrine reuptake inhibitors (SNRIs), selective serotonin reuptake inhibitors (SSRIs), or noradrenaline and specific serotonergic antidepressants (NaSSA). As all participants were hospitalized, diet, physical activity, and stress levels were institutionally standardized and thus not recorded in demographic data. These measures will be included in subsequent studies. Trained researchers obtained informed consent from all patients after receiving approval from the Ethics Committee of Hefei Fourth People’s Hospital.

### Event-related potential

Professional technicians specializing in electroencephalography within our hospital employed the American NicoletEDX electromyography evoked potential system to assess the patients. This took place in the electromyography room of our hospital from 9:00 to 11:30 a.m. upon admission. During the data collection process, the patients are usually seated upright in a comfortable chair, maintaining a relaxed state and focusing as much as possible. They are positioned approximately 1 m away from the computer screen, with the keyboard or button pad typically placed on their knees or in a tray. Electroencephalogram (EEG) signals are non-invasively recorded using electrodes, primarily made of silver/silver chloride or tin, which are attached to the scalp surface. Our electrode selection was primarily based on equipment limitations adhering to the 10/20 system and Cz’s superior signal-to-noise ratio compared to Pz in our sample. Additionally, while we analyzed all midline electrodes (Fz, Cz, and Pz), Cz demonstrated the most stable data for cognitive tasks, consistent with known P300 scalp distribution patterns. Thus, the electrodes were secured in an elastic nylon cap according to the International 10/20 System (American Neuromagnetic Society, 1994) (41), with Cz as the recording electrode, M2 (right ear) as the reference, and FPz (forehead center) as the ground lead. The signals were first amplified by scalp-mounted preamplifiers and then transmitted to a main amplifier (gain: ×10,000–50,000) for precise measurement. The parameters included the following: electrode impedance, <5 kΩ; bandpass filter, 0.5–100 Hz; and analysis time, 1,000 ms. The auditory stimuli comprised non-target (80% probability; 70 dB; 1,000 Hz) and target (20%; 90 dB; 2,000 Hz) tones presented randomly across two sessions; the responses were averaged for analysis. The electrodes are connected to a set of preamplifiers, which are located close to the participant’s scalp to provide sufficient initial amplification to transmit the weak signal to the main amplifier in the laboratory.

After enrollment, we collected the ERP parameters. MMN reflects the brain’s automatic processing function in response to diverse stimulus signals (42). N100 primarily indicates the integrity of the auditory conduction pathway and residual function of the primary auditory cortex, with minimal impact on higher cognitive assessment (43). The P200 component delineates the distinction between task-related stimuli and those unrelated to the task (44). The endogenous N200 is associated with selective response and also signifies the process of stimulus classification (45). P300 predominantly represents the initial processing of information stimuli in the brain, sensitively reflecting higher cognitive abilities and requirements for task processing speed (19, 46). We extracted the latency period of MMN, N1, P2, N2, P3a, and P3b (the horizontal linear distance from the onset of stimulation to the peak point of the maximum amplitude wave component) from ERP variables for analysis purposes.

### Polysomnography examination

Considering the first-night effect in sleep monitoring, we opted to analyze PSG data from patients on the second day. Throughout the night, all subjects were continuously monitored using the Embla N7000 device to assess neurophysiological and cardiopulmonary parameters. Sleep efficiency (SE) was calculated from the PSG data as a measure of sleep quality, with higher values indicating better sleep quality. SE is determined by the ratio of total sleep time [TST; TST is total sleep in minutes for all stages of sleep (stages 1, 2, and 3 non-REM and REM)] to time in bed [TIB; TIB begins with lights out and ends with lights on and is calculated in hours and minutes (also occasionally referred to as total bedtime time or TSP)], with higher values indicating improved sleep quality. Previous reviews have considered reduced SE in the PSG of MDD patients as a characteristic manifestation with high credibility (47). While difficulty falling asleep is commonly seen in various mental disorders, when it comes to evaluating the sleep patterns of MDD patients in clinical practice, sleep efficiency (SE) is a more appropriate measure. SE offers a more accurate depiction of the characteristics of sleep disturbances in MDD patients (47, 48). In normal individuals, SE values typically decrease with age, but they commonly remain above 90% for both youthful and middle-aged adults (49, 50).

### C-reactive protein

Peripheral cubital venous blood of 5 mL was collected from the patients upon admission, and the serum CRP level was determined by immunoturbidimetry. The blood samples were centrifuged at a low speed of 3,000 r/min for 10 min to harvest the upper layer of serum. The specimens were either promptly collected and analyzed within 1 h or stored at -80°C for further analysis. The level of CRP in the serum was analyzed using an automatic biochemical analyzer, following the manufacturer’s instructions.

### Scale assessment

The depression and anxiety symptoms of depressed patients were assessed using the 24-item Hamilton Depression Scale (HAMD) (51) and Hamilton Anxiety Scale (HAMA) (52). According to the HAMD scoring standard, a total score of 8~19 is mild depression, 20~34 is moderate depression, and ≥ 35 is severe depression. According to the HAMA scoring standard, a total score of more than 29 may indicate severe anxiety, more than 21 must have obvious anxiety, more than 14 may indicate anxiety, more than 7 may be anxiety, and less than 7 is not anxiety. All scale assessments are completed by trained researchers and entered into statistical software by professional statisticians.

### Sensitivity analysis

The SPSS 26.0 software package was utilized for data analysis, with count data being presented as frequency and percentage. The comparison of gender across different groups was conducted utilizing the *χ* ^2^ test. In cases where continuous variables did not follow a normal distribution, we described them using median (quartile range) [M(QR)] and conducted group comparisons using Mann–Whitney *U*-test. Measurement data were assessed with an independent sample *T*-test and presented as (x ± s). A repeated-measures ANOVA was employed to compare the differences in event-related potentials between the two groups with age, gender, BMI, education level, smoking habits, alcohol consumption, antidepressant usage, and use of benzodiazepine medication as covariates. Logistic regression analysis and mediation analyses were used to test the association between two variables and the mediating associations among three variables. Furthermore, we performed linear regression analysis on the latency of CRP and ERP in two groups of MDD. Moreover, to examine other variable-mediated relationships within the logistic regression analysis, we utilized the PROCESS plug-in tool. Finally, ordinary least squares path analysis evaluated both direct and indirect mediating effects of LnCRP on sleep efficiency and ERP-P300 latency. The statistical significance level was set at *P <*0.05. As CRP data showed skewness, CRP is measured in mg/L, and CRP was transformed to natural logarithms in the analysis (34).

## Results

### Demographic, clinical, and polysomnography characteristics

A total of 101 individuals diagnosed with MDD were enrolled and admitted to the Sleep Disorders Department at Hefei Fourth People’s Hospital between February 2021 and April 2023. They were categorized into two groups based on their sleep efficiency: one of which is a group with normal sleep efficiency (SE ≥90%; *N* = 45). The average age of the participants in the normal sleep efficiency group was 39.11 ± 13.78 years, consisting of 11 male and 34 female individuals. The low sleep efficiency group had an average age of 41.27 ± 12.93 years, including 23 male and 33 female individuals. Statistically significant differences were not observed in terms of age distribution, educational background, gender distribution, residential status, body mass index (BMI), alcohol consumption history, smoking history, scores on the Hamilton Depression Rating Scale (HAMD), scores on the Hamilton Anxiety Rating Scale (HAMA), use of antidepressant medication between, or use of benzodiazepines medication in these two groups (*P* > 0.05). However, a significant disparity was noted in C-reactive protein (CRP) levels (see **Table 1**).

**Table 1:** Comparison of traits in depressed individuals with low vs. normal sleep effectiveness (*n* = 101).

Characteristics	SE ≥ 90% (*n* = 45)	SE<90% (*n* = 56)	*Z*/*t*/*χ* ^2^	*P*
Age (year)^a^	39.11 ± 13.78	41.27 ± 12.93	-0.81	0.420
Gender, *n* (%)^b^			2.39	0.079
Male	11 (24.4)	23 (41.1)		
Female	34 (75.6)	33 (58.9)		
BMI (kg/m^2^)^a^	22.46 ± 3.28	23.74 ± 3.53	-1.87	0.064
Education level^b^			0.26	0.609
High school—below	24 (53.3)	27 (48.2)		
High school—above	21 (46.7)	29.7el—)		
Drink ≥1 time/week^b^	1 (2.2)	3 (5.4)	0.65	0.422
Smoking ≥1 branch/day^b^	3 (6.7)	5 (8.9)	0.18	0.676
Use of antidepressant medication^b^			0.398	0.941
SSRIs	19 (42.2)	24 (42.9)		
SNRIs	8 (17.8)	9 (16.0)		
NaSSA	4 (8.9)	7 (12.5)		
No use	14 (31.1)	16 (28.6)		
Use of benzodiazepines medication^b^	18 (40)	23 (41.1)	0.01	0.913
HAMD_24_ ^a^	31.80 ± 8.77	30.20 ± 9.24	0.89	0.377
HAMA^a^	21.23 ± 6.10	19.44 ± 6.26	1.41	0.161
CRP (mg/L)^c^	0.40 (0.55)	0.60 (0.88)	-2.29	0.022

SE, sleep efficiency; BMI, body mass index; SSRIs, selective serotonin reuptake inhibitors; SNRIs, serotonin norepinephrine reuptake inhibitors; NaSSA, noradrenergic and specific serotonergic antidepressant; HAMA, Hamilton Rating Scale for Anxiety; HAMD, Hamilton Rating Scale for Depression; CRP, C-reactive protein.

a *n*(%) description, and *χ* ^2^ test is employed for inter-group comparisons.

b $\bar{\text{x}}$ ± s description, and *t*-test is utilized for between-group comparisons.

c M(IQR) description, and Mann–Whitney *U*-test is applied for group comparisons.

### Comparison of indicators related to event-related potential

The latency differences of event-related potentials between the normal sleep efficiency group and the low sleep efficiency group were compared using an independent-samples *t*-test. No significant outliers were observed in the study data, and both groups exhibited distributions that approximated a normal distribution while meeting the assumptions of homogeneity of variance. The results indicated that the latencies of N2, P3a, and P3b in the low sleep efficiency group (239.16 ± 26.18, 292.27 ± 26.14, and 356.91 ± 27.32) were significantly higher than those in the normal sleep efficiency group (228.42 ± 27.78, 280.15 ± 24.63, and 336.46 ± 24). These differences reached statistical significance with effect sizes represented by *t*-values of -1.99 and -2.38 (*P* < 0.05) for N2 and P3a, respectively, and a *t*-value of -3.92 (*P* < 0.01) for P3b. After controlling for age and BMI through covariance analysis (ANCOVA), significant differences remained between groups regarding latencies of N2, P3a, and P3b [*F* = 4.50 (*P* = 0.036); *F* = 6.35 (*P* = 0.013); *F* = 15.22 (*P* < 0.001)]. No significant differences in latencies of MMN, N1, or P2 were observed between groups (*P* > 0.05) (see **Table 2**).

**Table 2:** Comparison of ERP-related indexes in MDD patients (*n* = 101).

ERP related indexes	SE ≥ 90% (*n* = 45)	SE < 90% (*n* = 56)	*t*	*F*	*P*
MMN (ms)	248.62 ± 34.98	248.41 ± 32.75	0.03	0	0.989
N1 (ms)	103.91 ± 18.27	103.88 ± 21.45	0.01	0.06	0.801
P2 (ms)	180.36 ± 18.67	184.08 ± 22.19	-0.90	0.68	0.411
N2 (ms)	228.42 ± 27.78	239.16 ± 26.18	-1.99*	4.50*	0.036
P3a (ms)	280.15 ± 24.63	292.27 ± 26.14	-2.38*	6.35*	0.013
P3b (ms)	336.46 ± 24.45	356.91 ± 27.32	-3.92**	15.22**	<0.001

The *t*-value represents the statistic of the *t*-test conducted between the two groups, while the *F*-value represents the statistic of analysis of covariance (ANCOVA) performed between the two groups after adjusting for age and BMI. Significance levels are denoted as *P <0.05 and **P <0.01.

### Direct association of SE with ERP latency

**Figure 1:**
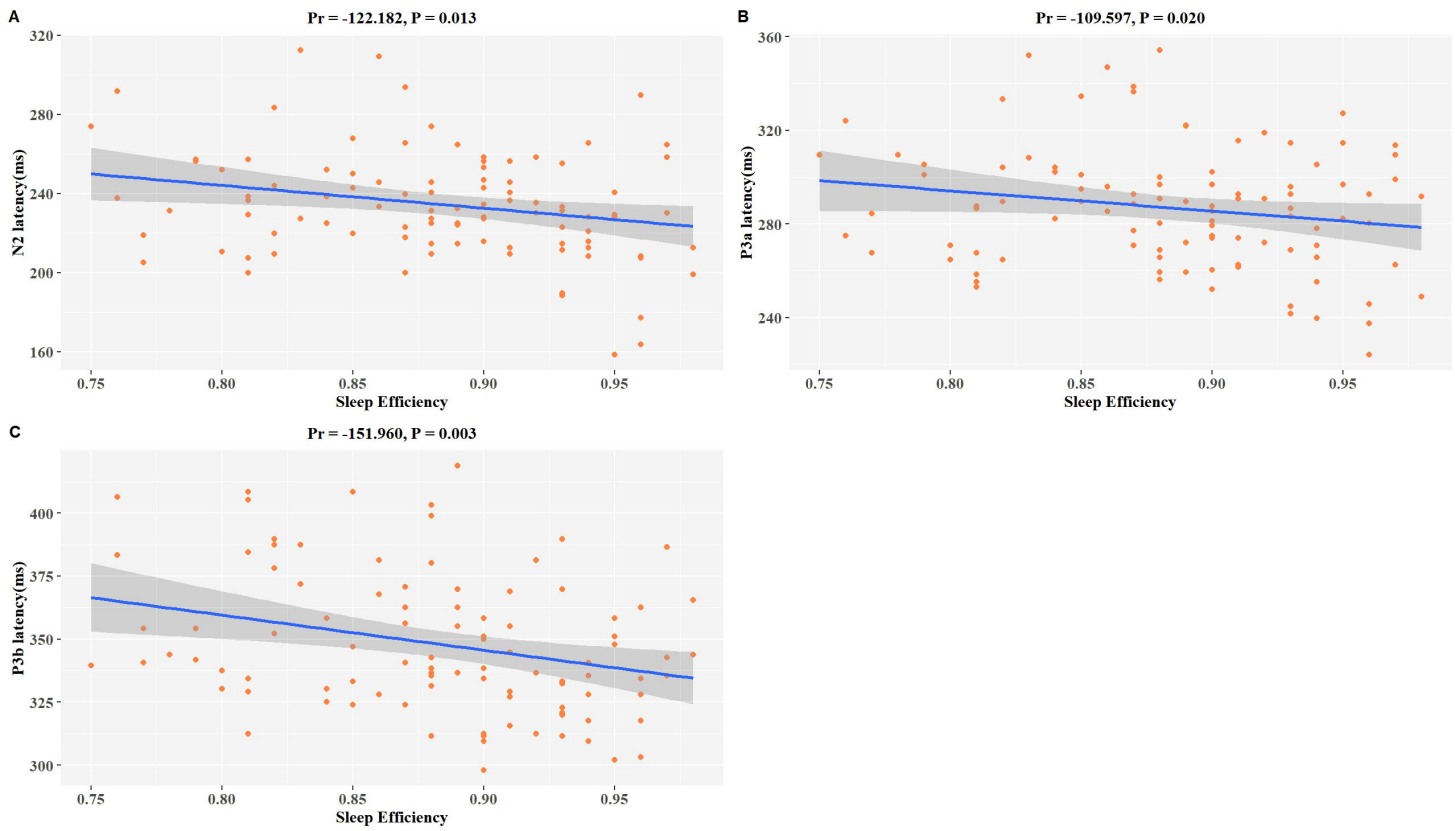
Associations between SE and P3b latency in MDD patients. The model was controlled for age, gender, BMI, education level, smoking habits, alcohol consumption, antidepressant usage, and use of benzodiazepine medication. **(A–C)** Scatter plots show a negative correlation trend between SE and changes in N2 latency, P3a latency, and P3b latency. The downward slope indicates negative correlations. pr is the partial correlation coefficient.

A significant correlation exists between sleep efficiency and ERP latency in patients diagnosed with MDD. After controlling for variables including age, gender, BMI, education level, smoking habits, alcohol consumption, and antidepressant usage, we discovered significant inverse associations between sleep efficiency and N2 latency (Pr = -122.182, *P* = 0.013), P3a latency (Pr = -109.597, *P* = 0.020), and P3b latency (Pr = -151.960, *P* = 0.003) within our study population (**Figures 1A–C**). These findings suggest that individuals with lower sleep efficiency display a noticeable cognitive impairment.

### Analysis of the correlation between SE, LnCRP, and LnCRP and ERP latency

**Figure 2:**
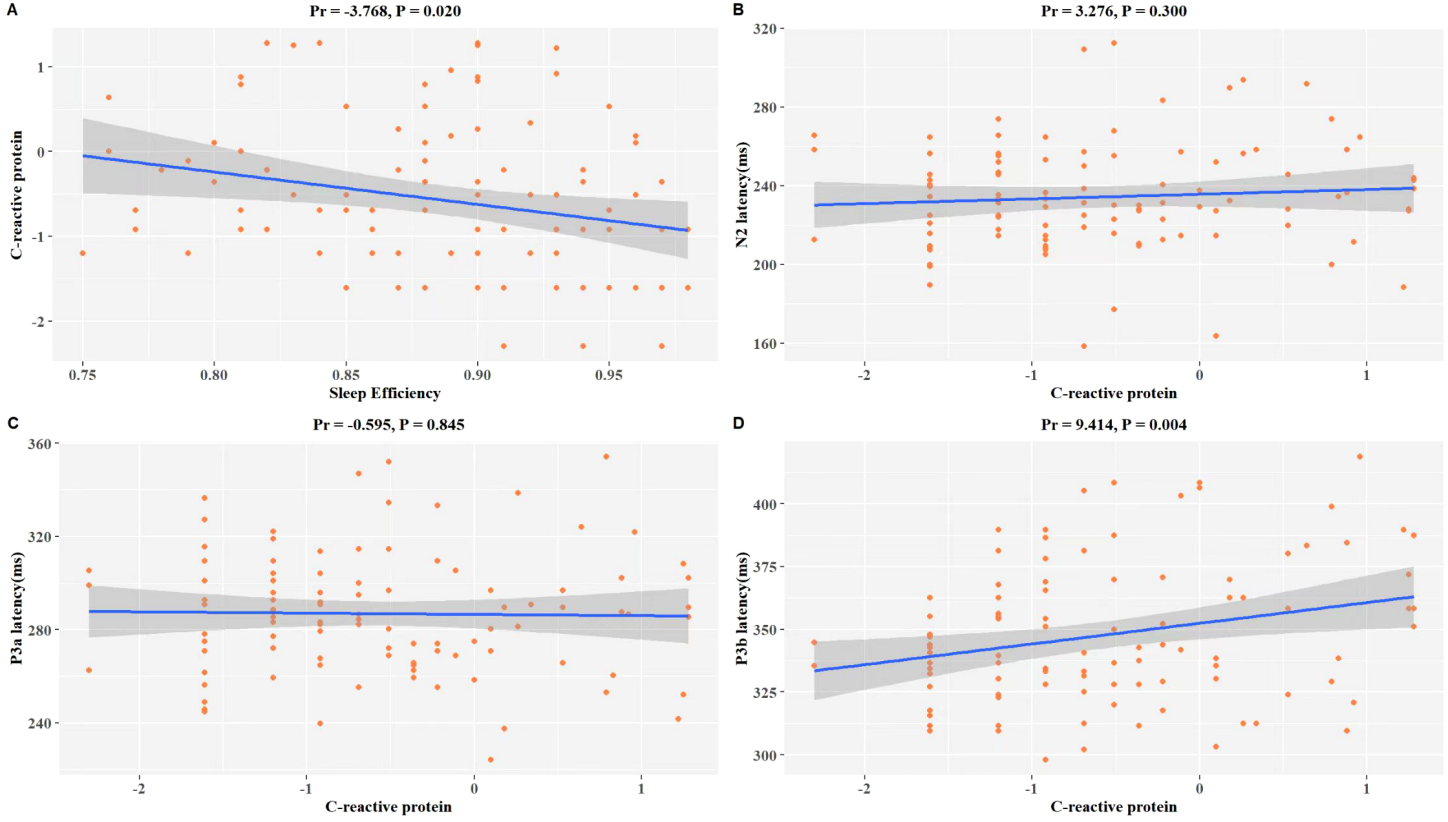
Association of LnCRP and sleep quality, and P300 latency of MDD. The model was controlled for age, gender, BMI, education level, smoking habits, alcohol consumption, antidepressant usage, and use of benzodiazepine medication. **(A)** Scatterplot of the relationship between LnCRP and SE. **(B)** Scatterplot of the relationship between LnCRP and N2 latency. **(C)** Scatterplot of the relationship between LnCRP and P3a latency. **(D)** Scatterplot of the relationship between LnCRP and P3b latency. Pr is the partial correlation coefficient. LnCRP and CRP were transformed to natural logarithms in the analysis.

A significant correlation exists among CRP and SE as well as ERP latency in patients with MDD. After adjusting for age, gender, BMI, education level, smoking habits, alcohol consumption, and antidepressant usage, we discovered significant inverse associations between SE and CRP (Pr = -3.768, *P* = 0.020) within our study population (**Figure 2A**). The results of the examination revealed as well a positive correlation between the level of CRP and the latency curves of N2 (Pr = 3.276, *P* = 0.300), P3a (Pr = -0.595, *P* = 0.845), and P3b (Pr = 9.414, *P* = 0.004) (**Figures 2B–D**). Specifically, there was a significant positive correlation with P3b, indicating that patients with elevated CRP levels may be at risk for a more severe cognitive impairment (**Figure 2D**).

### Correlation analysis of the latency period between CRP and ERP in various sleep efficiency groups

We performed additional experimental procedures to evaluate the consistency of our findings under different conditions. As shown in **Table 3**, after accounting for potential factors that may have an impact, controlling for age, gender, BMI, education level, smoking habits, alcohol consumption, antidepressant usage, and use of benzodiazepine medication, in individuals with low sleep efficiency MDD, the higher CRP levels were associated with increased levels of P3b latency [*β* with 95% CI: 15.202 (6.506, 23.897), *P* = 0.001], while this association was not observed in the normal sleep efficiency group (*β* = 0.984, *P* = 0.841).

**Table 3:** Comparison of P300-related indexes and CRP stratified by SE.

Variable	SE ≥ 90% (*n* = 45)		SE < 90% (*n* = 56)	
	*β* (95% CI)	*P*	*β* (95% CI)	*P*
N2 (ms)	-2.320 (-12.432, 7.792)	0.644	3.958 (-5.338, 13.253)	0.396
P3a (ms)	-6.437 (-14.899, 2.026)	0.132	2.960 (-6.017, 11.937)	0.510
P3b (ms)	0.984 (-8.919, 10.888)	0.841	15.202 (6.506, 23.8979	0.001**

The *t*-value represents the statistic of the *t*-test conducted between the two groups, while the *F*-value represents the statistic of analysis of covariance (ANCOVA) performed between the two groups after adjusting for age, gender, BMI, education level, smoking habits, alcohol consumption, antidepressant usage, and use of benzodiazepine medication. Significance levels are denoted as *P <0.05 and **P <0.01.

### Mediation by C-reactive protein

**Figure 3:**
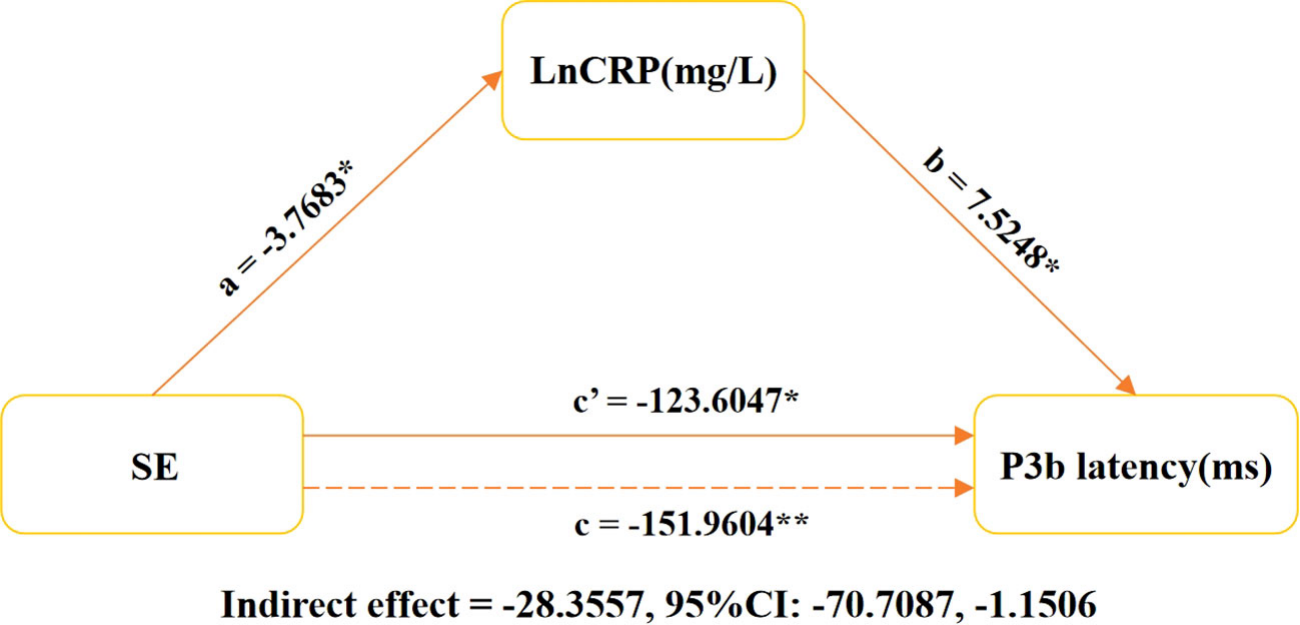
Relationship between latency of LnCRP, SE, and P3b in MDD patients. The graph illustrates the results of a mediation analysis on the relationship between sleep efficiency (SE), log-transformed C-reactive protein (CRP), and P3b latency. It includes estimates for mediated (a × b), direct (c′), and total effects, with LnCRP as the mediator. The models were controlled for age, gender, BMI, education level, smoking habits, alcohol consumption, antidepressant usage, and use of benzodiazepines medication. *P < 0.05, **P < 0.001. SE, sleep efficiency; P3b latency, latency of P3b component in event-related potentials. LnCRP and CRP were transformed to natural logarithms in the analysis. pr, partial coefficient; CI, confidence interval.

Additional comprehensive analysis is warranted; we employed a mediation model to examine the involvement of CRP in mediating the relationship between SE and P3b latency in individuals diagnosed with MDD. Our findings show that CRP has a significant mediating effect on the duration of P3b latency associated with SE (indirect effect = -28.3557, 95% CI: -70.7087, -1.1506; see **Figure 3**).

## Discussion

This study aimed to investigate the relationship between sleep efficiency, inflammation, and changes in cognitive function in patients with MDD. The study provides a comprehensive analysis of the associations of SE and serum CRP levels with ERP latency indicators. The findings indicated that alterations in sleep quality among MDD patients were linked to levels of serum CRP and P3b latency. Furthermore, a significant correlation was observed between serum CRP levels and ERP latency, specifically in patients with low sleep efficiency. Mediation analysis for patients with MDD suggested that sleep efficiency may be related to the P3b latency mediated by higher CRP. Our findings suggest a potential bidirectional relationship between elevated inflammatory markers, diminished sleep quality, and cognitive dysfunction in MDD patients. However, the observational nature of this study limits our ability to establish causal relationships.

Patients suffering from MDD regularly experience difficulties with sleep (53, 54), although not all individuals exhibit a noticeable reduction in sleep efficiency. Recent studies have indicated that self‐reported sleep appeared to reflect the sleep misperception commonly presented in persons with depressive and anxiety symptoms (55, 56). The results of retrospective self-report questionnaires may be influenced by potential bias and patients’ cognitive limitations as well as their pessimistic attitudes. Recently, objective assessment tools have received great attention and popularity in improving the detection and prevention of depression. Currently, PSG is considered the gold standard for sleep assessment tools (26, 27, 57). Additionally, receiving feedback from PSG results can assist in tailoring therapy specifically aimed at addressing sleep issues for the patients. Event-related potentials can overcome issues related to motivation and attention factors during measurement (17, 21, 58). The latest analysis suggests that P300, as a crucial component of the ERP, has indeed shown a significant clinical value in the study of cognitive dysfunction in MDD (8, 17).

Our findings support previous evidence (24, 36, 59) that MDD patients exhibit a negative correlation between sleep and cognitive dysfunction. Clinically, ERP is commonly utilized as a reliable indicator for evaluating the executive cognitive functions of patients, such as planning, decision-making, and problem-solving abilities (8, 27, 60). Consistent with previous studies (24, 48), there is a significant link between reduced sleep quality and impaired executive function. Low sleep efficiency struggles to maintain focus for extended periods and is distracted by external stimuli, which can hinder their ability to learn and perform tasks effectively daily (61, 62). Moreover, difficulties in memory capacity can cause significant inconvenience in both personal and professional aspects of life (5, 40, 63). Getting enough sleep plays a crucial role in improving cognitive function for patients with MDD (9, 26, 36). Research in the field of mood disorders consistently shows that sleep affects cognitive function regulation through various ways and pathways (8, 9, 26), poor sleep quality exacerbates cognitive decline, and early intervention can improve patient prognosis and cognitive recovery (7, 10). Our study offers a fresh perspective: improving the sleep quality of people with MDD could be a potential strategy to reduce their cognitive decline.

Poor sleep quality not only directly affects cognitive function but also influences inflammatory factor levels. Our findings demonstrate that reduced sleep efficiency is associated with elevated serum C-reactive protein (CRP) levels. Impaired sleep may contribute to increased inflammatory markers (59, 60), and prolonged inflammatory responses could play a role in the onset and progression of mood disorders (2, 27, 35). In patients with MDD, heightened levels of peripheral inflammatory markers may correlate with the severity of specific clinical symptoms (28, 61. Accumulating evidence shows that sleep quality affects inflammatory factor regulation, potentially leading to health issues (30–32). In short, these results suggest that sleep disruptions could lead to increased levels of inflammation markers. Note the significant correlation between sleep efficiency in patients with MDD and serum levels of CRP, which aligns with previous research demonstrating the impact of sleep disturbances on elevated inflammatory markers in animal models (64, 65). Our results align with prior studies indicating a possible link between poor sleep quality and increased inflammation in individuals experiencing depression (66, 67). The somatic symptoms of MDD may have a stronger link to inflammatory responses than affective symptoms (35, 68, 69), and improving sleep quality can reduce systemic inflammation in patients with MDD (67, 70). Low-level systemic inflammation may be involved in various neuropsychiatric disorders that affect mood and cognitive function (30, 39, 40). Our study discovered that the association between inflammation and impaired cognitive function in individuals with MDD aligns with earlier research results (30, 31, 39, 67, 71). The presented evidence illuminates the intricate interplay of sleep, biomarker activation, and cognitive performance in patients with MDD. Research has found that the decline of cognitive and executive functions among individuals with obstructive sleep apnea (OSA) is associated with inflammation, but this association could be alleviated by reducing serum CRP (72, 73). Reversing inflammation could be a potential strategy to address cognitive decline with MDD.

Previous research on the neurobiology of MDD accompanied by sleep disturbances has mainly focused on factors such as metabolomics, changes in hormone levels, alterations in neurotransmitters, genetics, and epigenetics. Limited investigation has been conducted on the associations among serum CRP levels (a marker of inflammation) and both sleep quality and cognitive function (74, 75). Various biomarkers exert their effects through distinct signaling pathways, leading to diverse impacts on the regulation of neurocognitive function (76, 77). This variation may result from the varying levels of cognitive impairment among the subjects. Sleep regulates gene expression in the sympathetic nervous system and hypothalamic–pituitary–adrenal axis, leading to fluctuations in inflammation levels that affect cognitive function (30, 37, 78). Certain research examines the connection between sleep, inflammation, and cognitive function in patients with MDD as well as the effects of atypical sleep duration on response time and visual memory (30, 39, 54, 79). Previous studies have found that changes in white blood cells can affect cognitive function during sleep (71), and there is a significant association between systemic inflammation and nighttime awakening and dementia occurrence (74). Consistent with our findings, MDD with lower SE correlates with higher CRP levels, which may be related to the severity of cognitive impairment (74, 79). Serum CRP levels are correlated with SE and neurocognitive function. Among them, CRP may mediate the association between SE and impaired cognitive function. The improvement of SE may help reduce the level of serum CRP, thereby helping to prevent adverse neurocognitive changes.

Consistent with other studies, one of the main reasons for the poor prognosis of patients with MDD cognitive dysfunction is their difficulty in recovery, and currently, antidepressant drugs have limited ability to improve cognitive function. Our study has identified distinct alterations in cognitive function and levels of the inflammatory marker CRP in individuals with major depressive disorder (MDD) and poor sleep quality. Additionally, our study further elucidates the sequential relationships among sleep, CRP, and cognition, highlighting their close interdependence and emphasizing the complexity of connections in the prognosis of MDD. These results shed light on potential biological pathways linking sleep and cognitive function, offering valuable insights for the early detection and management of cognitive impairment in patients with depression.

### Strengths and limitations

Previous studies hint at a connection between sleep efficiency (SE) and cognitive function, and our research indicates that CRP plays a key role in this link. We utilized objective measurements for SE and event-related potential P300 indicators. For individuals with MDD (8, 17, 58), event-related potential measurements are more sensitive in evaluating cognitive function and detecting differences compared to scale assessments, enhancing the reliability and accuracy of our data. Future research should explore the causes and directions of these connections. Through longitudinal studies, the immunological mechanisms behind neurocognitive decline in individuals with MDD will be clarified.

This approach shows promise to address cognitive impairment in MDD; however, there is currently limited research on this topic within the field of MDD. There are, however, certain limitations to this study. As it is a cross-sectional study lacking functional intercept indicators, it is impossible to ascertain whether CRP mediates the causal relationship between sleep efficiency and impaired cognitive function. Consequently, longitudinal studies are necessary to further validate the role of CRP as a mediating variable, which still requires improvement. The small sample size may restrict the detection of changes in serum CRP levels and hinder the identification of potential associations among depression, cognition, and sleep. Additionally, potential confounding factors such as disease episode frequency, illness duration, and treatment history were not fully accounted for, which may influence the observed relationships. Future studies should further refine the collection and analysis of such clinical variables. Due to the global health crisis at that time and ethical considerations, we are unable to recruit a normal control group for PSG and ERP data collection in the ward. Future research should consider incorporating a robust control cohort. While polysomnography is utilized to mitigate the first-night effect in clinical practice, its effectiveness for normal controls in a hospital setting is limited (54, 61, 71). Subsequent studies could investigate alternative sleep measurement methods, such as activity monitoring. A larger sample size is essential for more precise outcomes and reliable data for clinical prognosis.

## Conclusion

We found a robust association of the poorer sleep quality with the higher levels of CRP and cognitive function decline in patients with MDD. The levels of inflammatory factors may mediate the relationship between sleep efficiency and cognitive decline. Improved sleep quality and anti-inflammatory agents or diet (micronutrient supplementation) could prevent cognitive decline in patients with MDD. Further randomized controlled intervention trials are needed.

## Data Availability

The raw data supporting the conclusions of this article will be made available by the authors, without undue reservation.
